# The influence of depression symptoms on postmorbid adaptation of patients after SARS-CoV-2 infection

**DOI:** 10.1192/j.eurpsy.2023.449

**Published:** 2023-07-19

**Authors:** A. Barkhatova, S. Sorokin

**Affiliations:** Department of endogenous mental disorders and affective states, Mental health research center, Moscow, Russian Federation

## Abstract

**Introduction:**

The study discusses the possibility of assessing postmorbid adaptation in depressed patients who had SARS-CoV-2 infection, within the framework of a multimorbidity model. This model takes into account not only mental, but also somatic clusters and allows to identify non-randomly occurring, related conditions and their impact on the ability to recover.

**Objectives:**

The aim of the study is evaluation of the impact of psychosomatic and anxiety symptoms of depression on the formation of postmorbid adaptation in a group of patients who underwent outpatient SARS-CoV-2 infection.

**Methods:**

The data of 54 patients aged 25-55 years with a current depressive episode who underwent outpatient SARS-CoV-2 infection were analyzed. We used the Hamilton depression rating scale (HDRS); the somatic condition was assessed using a general assessment of the patient’s function limitation at the stage of convalescence. A linear regression analysis was performed to assess the association of psychosomatic and anxiety symptoms with somatic condition in SARS-CoV-2 infection, regardless of other factors.

**Results:**

An association was established in patients with SARS-CoV-2 infection showing signs of psychosomatic and anxiety symptoms of depression. Patterns of somatic and anxiety symptoms were independently associated with impaired postmorbid adaptation, other manifestations of depression did not show such association. This fact raises the question of whether depression syndrome is really associated with an impaired postmorbid adaptation in the postcovid period, or only certain specific symptoms of somatic anxiety explain the low readaptation capacity in this group of patients.

**Conclusions:**

Identification of clusters of symptoms associated with an impact on the full recovery possibilities in the SARS-CoV-2-postmorbid period can significantly help in providing high-quality and targeted psychopharmacological care.

**Disclosure of Interest:**

None Declared

